# Automated follicle measurement and microvascular imaging accuracy in assessing diminished ovarian reserve via 3D ultrasound

**DOI:** 10.3389/fmed.2026.1690928

**Published:** 2026-04-29

**Authors:** Dan Hu, Ke Hong Hao, Qi Yue Li, Min Ren, Yun Qi Chen

**Affiliations:** Ultrasound Department, Shanghai Key Laboratory of Maternal Fetal Medicine, Shanghai Institute of Maternal-Fetal Medicine and Gynecologic Oncology, Shanghai First Maternity and Infant Hospital, School of Medicine, Tongji University, Shanghai, China

**Keywords:** automated follicle measurement, diminished ovarian reserve, superb micro-vascular imaging, three-dimensional, ultrasonography

## Abstract

**Purpose:**

This study aimed to investigate the diagnostic values of automated follicle measurement and superb microvascular imaging using transvaginal three-dimensional (3D) ultrasound for diminished ovarian reserve (DOR) in women of reproductive age.

**Methods:**

This retrospective study included women who underwent ovarian reserve function assessment at the Affiliated Obstetrics and Gynecology Hospital of Tongji University between November 2022 and June 2023. The participants were categorized into the DOR and normal groups based on the anti-Müllerian hormone (AMH) and follicle-stimulating hormone (FSH) levels.

**Results:**

This study included 109 participants: 36 with diminished ovarian reserve (DOR) and 73 with normal ovarian function. Significant differences emerged between the groups in age, body mass index, AMH, FSH, the FSH/LH ratio, and estradiol (all *p* < 0.05). Transvaginal 3D ultrasound parameters also differed significantly, including antral follicle count (AFC), total ovarian volume (OV), the stroma-to-antrum ratio (S/A), vascularization index (VI), flow index (FI), vascularization-flow index (VFI), and ovarian interstitial artery peak and end-diastolic velocities (PSV, EDV) (all *p* < 0.05). The efficacy of the ultrasound–serum combined index in diagnosing DOR (AUC = 0.995, 95% CI: 0.949–1.000) is superior to that of the multi-parameter ultrasound index (AUC = 0.973, 95% CI: 0.914–0.996), AFC (AUC = 0.965, 95% CI: 0.902–0.993), and AMH (AUC = 0.974, 95% CI: 0.916–0.996). According to the ROC curve analysis, when selecting the cutoff value based on the highest sensitivity, the optimal cutoff for AFC was 6.5. After including the age variable, least absolute shrinkage and selection operator (LASSO) regression identified AFC, stromal OV, total VI, total FI, and EDV as optimal variables. Among them, AFC had the highest weight coefficient. The OR values of AFC (OR = 0.596, 95% CI: 0.442–0.804, *p* < 0.001) and total FI (OR = 0.853, 95% CI: 0.737–0.988, *p* = 0.033) in the multivariate analysis are statistically significant. Decision curve analysis (DCA) demonstrated that the PRE_LASSO model consistently outperformed the PRE_AFC model in net benefit across most threshold ranges. The scatter plot of net reclassification improvement (NRI) showed that most case patients had increased risk, while control patients had reduced risk, indicating that the PRE_LASSO model exhibits superior clinical application value and risk stratification capability.

**Conclusion:**

This study identified AFC as the key predictor factor for DOR, with an AUC of 0.965 (95% CI: 0.902–0.993), demonstrating high diagnostic value. These results underscore the promising potential of transvaginal 3D ultrasound techniques to enhance the precision of ovarian reserve assessments.

## Introduction

Diminished ovarian reserve (DOR) refers to a reduction in the number and quality of oocytes in the ovaries, which significantly affects a woman’s fertility potential (1). DOR not only leads to infertility but also impacts overall reproductive health and quality of life. Early prediction, diagnosis, and treatment of DOR are essential to restore ovarian function and improve pregnancy rates (2). Current methods for assessing ovarian reserve include measurements of age, basal hormone levels, anti-Müllerian hormone (AMH), ovarian volume, and the antral follicle count (3, 4). While hormone detection provides valuable information, each method has its limitations. AMH levels can vary based on individual physiology and external factors, and basal hormone levels fluctuate throughout the menstrual cycle (5). Ultrasound examination is widely applied and promoted in clinical practice due to its advantages, such as simplicity, non-invasiveness, dynamic nature, and reproducibility. However, traditional two-dimensional ultrasonographic assessments may not capture the full complexity of ovarian morphology and vascularization, nor can they automatically and accurately obtain the sinus follicle count, leading to potential inaccuracies in evaluating ovarian reserve (1, 6). These limitations highlight the need for more reliable and comprehensive diagnostic tools.

In recent years, with the rapid development of three-dimensional (3D) ultrasound technology, it has become a promising tool for ovarian reserve assessment (7). The 3D ultrasound provides detailed images of ovarian structures, allowing for more accurate measurements of ovarian volume and antral follicle count (8). The ability to visualize the ovaries in three dimensions offers a more comprehensive assessment of ovarian morphology, which is crucial for accurately diagnosing DOR. Moreover, 3D ultrasound can be combined with superb microvascular imaging to evaluate blood flow within the ovaries, providing additional insights into ovarian health that are not accessible with conventional 2D ultrasound (4).

The objective of this study is to assess ovarian reserve function using automatic follicle measurement and superb microvascular imaging in transvaginal 3D ultrasound and to explore its clinical diagnostic and application value for DOR in women of reproductive age.

## Materials and methods

### Study design and participants

A retrospective study was conducted on women who underwent ovarian reserve function assessment due to infertility at the Center for Reproductive Medicine, Shanghai First Maternity and Infant Hospital of Tongji University from November 2022 to June 2023. As this study is retrospective, informed consent was waived. The study protocol was reviewed and approved by the Ethics Committee of Affiliated Obstetrics and Gynecology Hospital of Tongji University (Approval No. 2024-070). The inclusion criteria were as follows: (1) women of reproductive age (20–49 years); (2) presence of bilateral ovaries; and (3) no history of ovarian surgery. The exclusion criteria were as follows: (1) presence of organic lesions of the ovaries; (2) ovarian dysfunction or premature ovarian failure resulting from bilateral or unilateral ovarian surgery; (3) presence of other gynecological tumors or endocrine disorders affecting metabolism and secretion, such as hyperthyroidism, hypothyroidism, pituitary tumors, insulin resistance, hyperprolactinemia, and polycystic ovary syndrome; (4) presence of severe cardiovascular or cerebrovascular diseases; (5) use of hormonal medications within the past 3 months; (6) uterine adhesions resulting from uterine surgery; and (7) poor-quality ultrasound images of bilateral ovaries.

### Transvaginal 3D ultrasound

The ultrasound examinations were conducted using the MaiRui Resona R9 Pro and R9 Elit color Doppler ultrasound diagnostic instrument, equipped with a transvaginal 3D intracavitary ultrasound probe (model DE10-3WU) with a frequency range of 4–9 MHz. All examinations were performed by a senior ultrasound physician with more than 5 years of experience in obstetric and gynecological ultrasound. The assessments were carried out on the second to fourth day of the menstrual cycle with the patient in the lithotomy position and an empty bladder. The transvaginal probe, covered with a condom, was gently inserted into the vaginal introitus to reach the vaginal fornix. Initially, two-dimensional ultrasound was used to observe the size, morphology, and internal echoes of the uterus and ovaries. Following this, the 3D ultrasound mode was selected.

**Figure 1:**
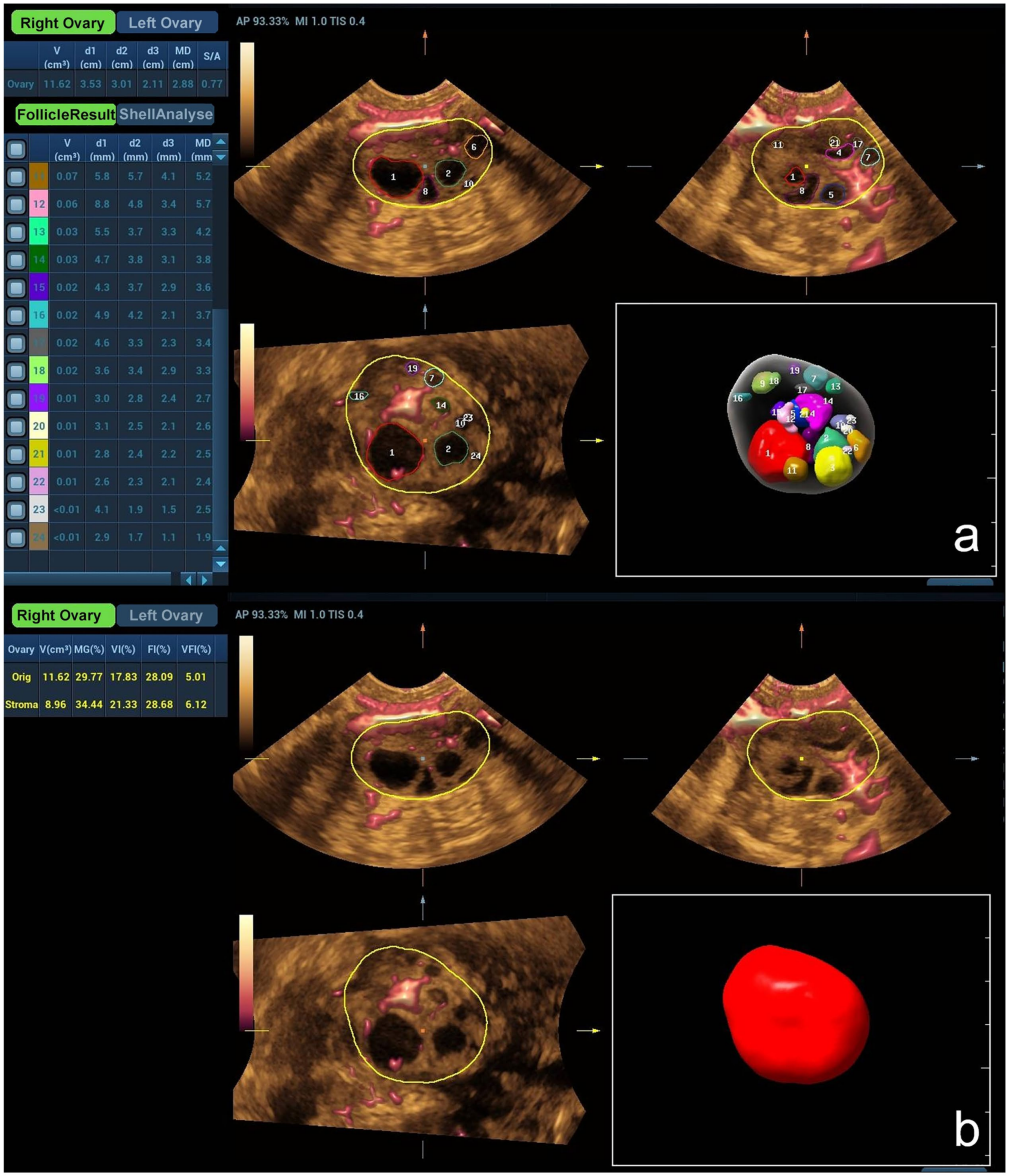
**(a)**Transvaginal three-dimensional automatic follicle measurement and ultra-microvascular flow imaging of right ovary follicles in a normal patient. **(a)** Automatic follicle measurement shows multiple antral follicles (numbered 1–24) in the right ovary (yellow outline), and the left panel lists each follicle’s indicators (OV, diameter d1/d2/d3, mean diameter MD, etc.). Color Doppler imaging shows the vascular distribution around the follicles. **(b)** Shows the ultra-microvascular flow and three-dimensional volume imaging (red structure) of the same right ovary (yellow outline), and the left panel lists origin and stromal OV, MG, VI, FI, and VFI.

In the 3D mode, automatic follicle measurement and superb microvascular imaging technology were utilized. The ovaries are automatically scanned by manually adjusting the sampling frame according to the size of the ovaries. Post-scan, the system displayed parameters including total and stromal ovarian volume (OV), antral follicle count (AFC), stromal-to-antrum ratio (S/A), total and stromal mean gray (MG), total and stromal vascularization index (VI), total and stromal flow index (FI), and total and stromal vascularization flow index (VFI). All identified follicles are displayed in different color codes according to their volume size and listed sequentially, which can be manually edited to adjust for identification bias in the measurement (Figure 1).

**Figure 2:**
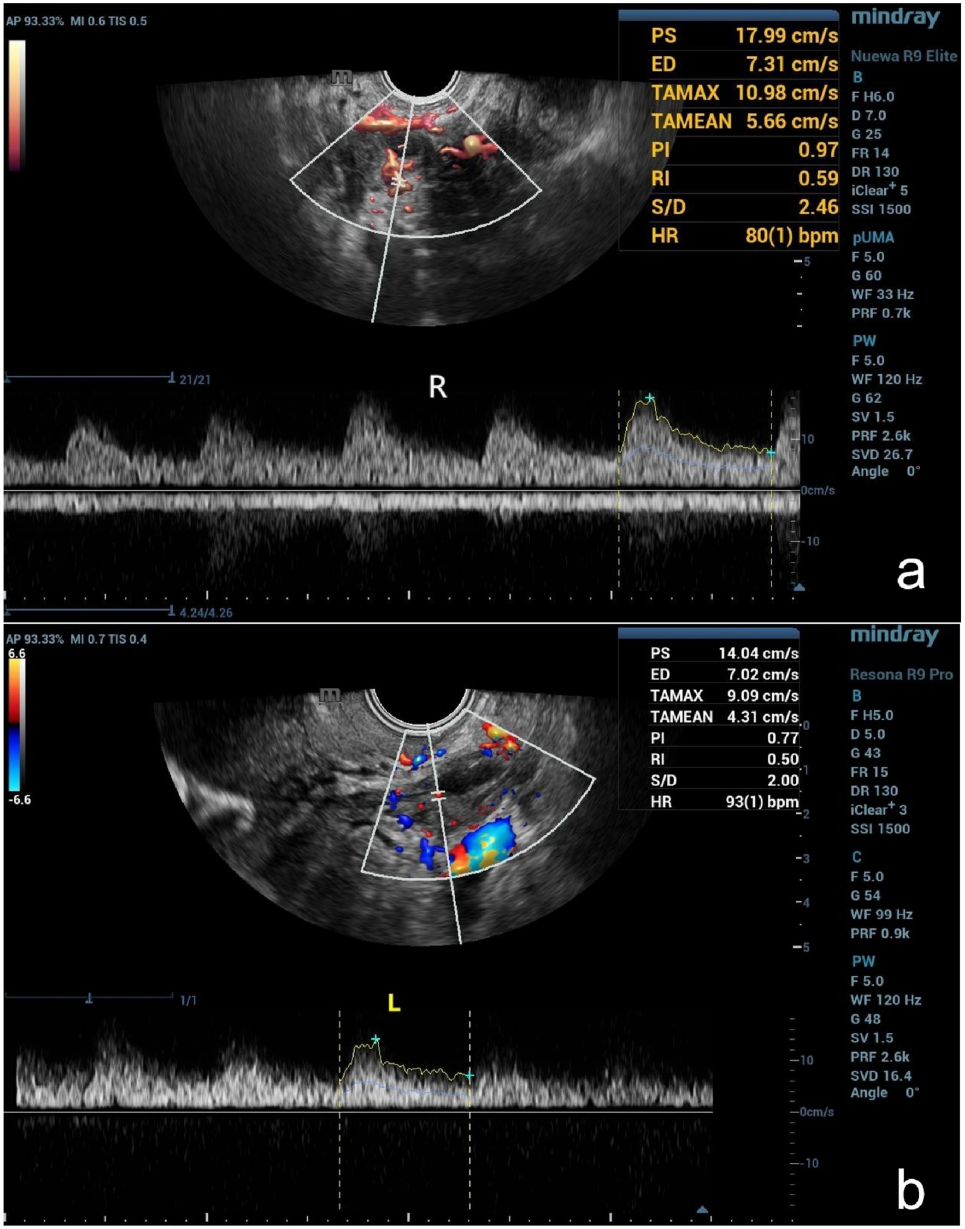
**(a)** Spectral measurement of the interstitial artery in the right ovary in the same normal patient and **(b)** blood flow spectrum measurement in the left ovarian interstitial artery in a patient with DOR.

Color Doppler sampling frames were strategically placed in the ovarian medulla to visualize the ovarian interstitial arteries. The sampling volume was placed on the larger coarse interstitial artery, and their peak systolic flow velocity (PSV), end-diastolic flow velocity (EDV), pulsatility index (PI), and resistance index (RI) were measured (Figure 2). The AFC, representing the sum of antral follicles in both ovaries, was recorded for statistical analysis, while all other ultrasound parameters were averaged from both ovaries.

### Data collection

Patient data, including age, body mass index (BMI), and hormone levels, were collected from medical records. On the morning of the second to fourth day of their menstrual cycle, all study subjects provided venous blood samples from the cubital vein. To minimize potential temporal bias, the ultrasound examinations and hormonal assessments were conducted within 24–48 h. Serum levels of AMH, follicle-stimulating hormone (FSH), luteinizing hormone (LH), and estradiol (E2) were measured using a fully automated biochemical analyzer. The FSH/LH ratio was subsequently calculated. The diagnostic criteria for DOR were defined as follows: (1) AMH <1.1 ng/mL (9) and (2) basal FSH ≥10 IU/L for two consecutive menstrual cycles (the interval between measurements exceeds 4 weeks) (10). All participants were categorized into the DOR or normal groups accordingly.

### Statistical analysis

Statistical analysis was performed using R 4.5.2 software. Continuous variables with normal distribution were expressed as mean ± standard deviation (SD), and comparisons between groups were made using independent sample *t*-tests. Continuous variables with skewed distributions were expressed as M (P25, P75), and non-parametric tests were used for group comparisons. Univariate and multivariate logistic regression analyses were performed on the indicators screened by LASSO analysis. The independent influencing factors associated with DOR were identified through univariate and multivariate logistic regression analyses. Receiver operating characteristic (ROC) curves, DCA curves, and NRI scatter plots were plotted, and metrics such as the area under the ROC curve (AUC) and NRI were calculated to evaluate the diagnostic value of different models. A two-sided *p*-value of less than 0.05 was considered statistically significant.

## Results

### Clinical characteristics and serum hormone levels between groups

This study included a total of 109 participants, ranging in age from 18 to 47 years, with a mean age of 33.89 ± 5.86 years. Among them, 36 cases were diagnosed with DOR, with ages ranging from 28 to 46 years and a mean age of 37.50 ± 4.31 years. The remaining 73 cases constituted the normal group, with ages ranging from 18 to 47 years and a mean age of 32.11 ± 5.72 years. There were statistically significant differences between the two groups in terms of age (*p* < 0.001), BMI (*p* = 0.014), AMH (*p* < 0.001), FSH (*p* < 0.001), FSH/LH ratio (*p* < 0.001), and E2 (*p* = 0.014). However, the difference in LH levels was not statistically significant (*p* = 0.902) (Table 1).

**Table 1:** Comparison of clinical characteristics of participants.

Index	Normal group (*n* = 73)	DOR group (*n* = 36)	*p*
Age (years)	32.11 ± 5.72	37.50 ± 4.31	<0.001
BMI (kg/m^2^)	22.00 (21.10, 22.00)	22.31 (21.55, 23.02)	0.014
AMH (ng/mL)	4.14 (3.15, 6.82)	0.36 (0.18, 0.86)	<0.001
FSH (IU/L)	6.74 (5.85, 7.78)	12.16 (9.66, 22.91)	<0.001
LH (IU/L)	4.43 (3.04, 6.22)	3.47 (2.86, 9.80)	0.902
FSH/LH	1.54 (1.08, 2.01)	2.90 (1.78, 3.86)	<0.001
E2 (pg/mL)	0.92 (0.60, 16.85)	21.81 (0.80, 40.10)	0.014

BMI, body mass index; AMH, anti-Müllerian hormone; FSH, follicle-stimulating hormone; LH, luteinizing hormone; E2, estradiol.

### Ultrasound examination parameters between the two groups

Transvaginal 3D ultrasound follicle automatic measurement and superb microvascular imaging detected significant differences between the two groups in all parameters except for stromal MG. Specifically, parameters such as AFC (*p* < 0.001), S/A (*p* < 0.001), total OV (*p* < 0.001), stromal OV (*p* < 0.001), total MG (*p* = 0.014), total VI (*p* = 0.004), stromal VI (*p* = 0.002), total FI (*p* = 0.001), stromal FI (*p* = 0.002), total VFI (*p* = 0.004), and stromal VFI (*p* = 0.002) exhibited statistically significant differences between the two groups. Additionally, there were statistically significant differences in ovarian interstitial artery PSV (*p* = 0.002) and EDV (*p* = 0.001) between the two groups, while no statistically significant differences were observed in PI (*p* = 0.687) and RI (*p* = 0.929) (Table 2).

**Table 2:** Comparison of ultrasound parameters between the normal and DOR groups.

Index	Normal group (*n* = 73)	DOR group (*n* = 36)	*p*
AFC	24.00 (15.00, 36.00)	5.00 (2.00, 8.25)	<0.001
S/A	0.86 (0.82, 0.90)	0.93 (0.86, 0.98)	<0.001
Total OV (cm^3^)	6.85 (5.81, 8.31)	4.03 (2.87, 4.72)	<0.001
Stromal OV (cm^3^)	5.81 (5.05, 7.38)	3.64 (2.65, 4.23)	<0.001
Total MG (%)	32.18 ± 3.98	34.37 ± 4.94	0.014
Stromal MG (%)	34.63 ± 3.84	35.68 ± 4.77	0.251
Total VI (%)	12.32 (7.48, 19.64)	8.09 (3.12, 12.18)	0.004
Stromal VI (%)	14.23 (8.54, 22.59)	8.76 (3.58, 13.34)	0.002
Total FI (%)	25.55 (24.09, 28.16)	23.78 (21.16, 25.74)	0.001
Stromal FI (%)	25.88 (24.25, 28.23)	23.84 (21.14, 26.04)	0.002
Total VFI (%)	3.12 (1.94, 5.80)	2.24 (0.72, 3.59)	0.004
Stromal VFI (%)	3.60 (2.15, 6.57)	2.37 (0.78, 3.79)	0.002
PSV (cm/s)	16.24 (13.55, 19.82)	13.02 (9.76, 17.25)	0.002
EDV (cm/s)	7.16 (5.80, 8.43)	5.60 (4.44, 6.77)	0.001
PI	0.89 (0.75, 1.04)	0.88 (0.79, 1.17)	0.687
RI	0.56 ± 0.09	0.56 ± 0.1	0.929

AFC, antral follicle count; S/A, stroma-to-antrum ratio; OV, ovarian volume; MG, mean gray; VI, vascularization index; FI, flow index; VFI, vascularization flow index; PSV, peak systolic velocity; EDV, end-diastolic velocity; PI, pulsatility index; RI, resistance index.

### Diagnostic value of age, BMI, various serum indicators, various independent ultrasound indicators, multi-parameter ultrasound indicator, and ultrasound serum combined indicator for ovarian reserve function

**Figure 3:**
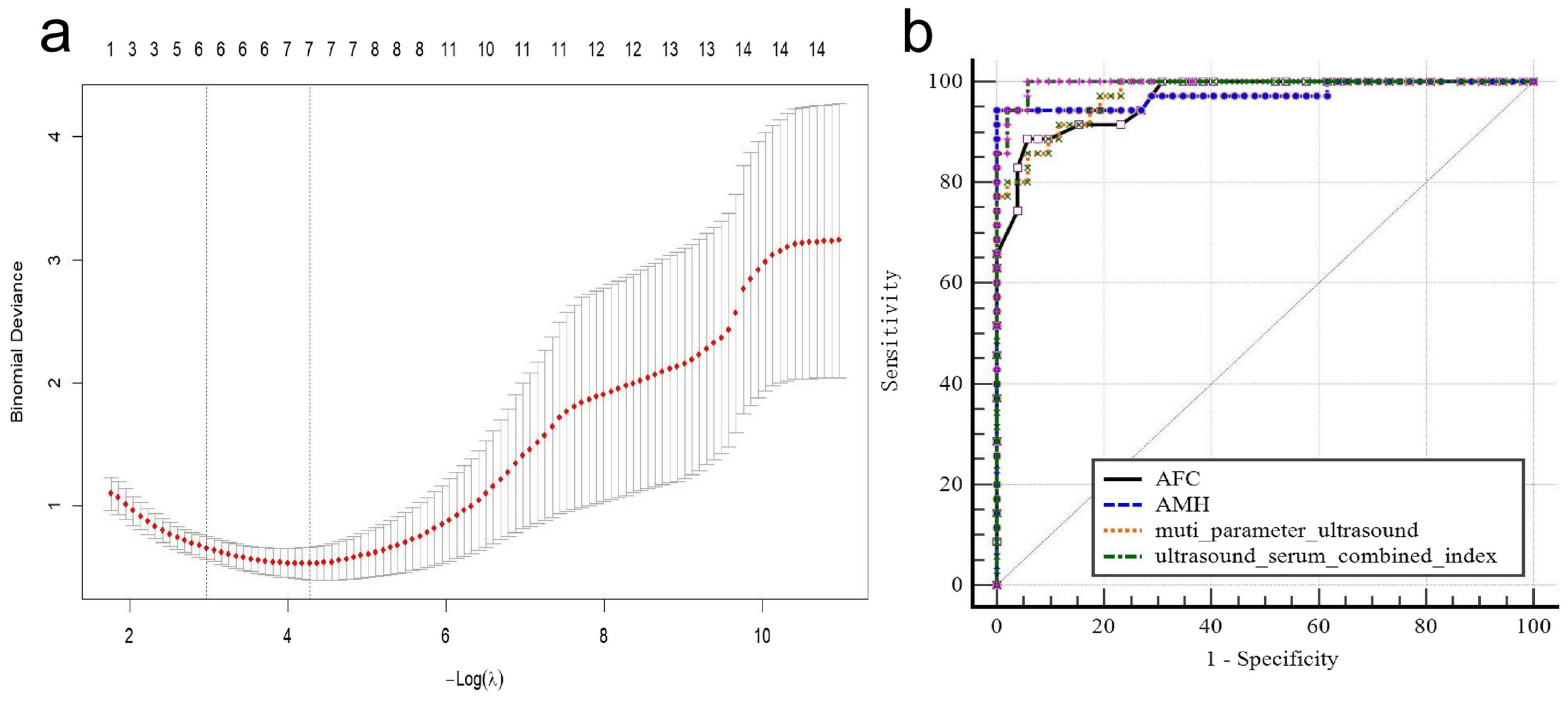
**(a)** LASSO regression analysis; **(b)** ROC curve of AFC, AMH, multi-parameter ultrasound, and ultrasound–serum combined index for diagnostic value of ovarian reserve function.

Among all ultrasound indicators, the area under the AFC curve showed the highest value (AUC = 0.965, 95% CI: 0.902–0.993), with a sensitivity of 88.89% and a specificity of 93.15%. The diagnostic efficacy of the multi-parameter ultrasound indicator (AUC = 0.973, 95% CI: 0.914–0.996, sensitivity 91.67%, specificity 89.04%) was superior to AFC but comparable to AMH (AUC = 0.974, 95% CI: 0.916–0.996, sensitivity 94.44%, specificity 100%). The ultrasound–serum indicator demonstrated optimal diagnostic performance for DOR (AUC = 0.995, 95% CI: 0.949–1.000, sensitivity 100%, specificity 94.23%). According to the ROC curve analysis, when selecting the cutoff value based on the highest sensitivity, the optimal cutoff of antral follicle count was 6.5, the optimal cutoff of total OV was 3.27 cm^3^, and the optimal cutoff of stromal OV was 1.81 cm^3^ (Table 3). The ROC curve (Figure 3b) illustrated the diagnostic value of AFC, AMH, multi-parameter ultrasound indicator, and ultrasound–serum combined indicator for ovarian reserve function. The DeLong test was used to compare the differences in the area under the ROC curve (AUC) between different indicators. The results showed that there were no statistically significant differences between AFC and AMH or multi-parameter ultrasound, while there was a statistically significant difference between AFC and the ultrasound–serum combined indicator (*p* = 0.046) (Table 4).

**Table 3:** Diagnostic value of age, BMI, serum indicators, ultrasound indicators, and combined indicators for ovarian reserve function.

Index	The optimal cut-off	Youden’s index	AUC	95% CI	Sensitivity (%)	Specificity (%)	Kappa value	Positive predictive value (%)	Negative predictive value (%)
Age	33.00	0.518	0.780	0.691–0.854	86.11	65.75	0.454	55.36	90.57
BMI	22.00	0.406	0.643	0.546–0.733	63.89	76.71	0.394	57.50	81.16
AMH	1.08	0.944	0.974	0.916–0.996	94.44	100.00	0.952	100.00	96.30
FSH	8.55	0.747	0.847	0.754–0.915	80.56	94.23	0.760	90.63	87.50
FSH/LH	2.11	0.455	0.754	0.650–0.839	66.67	78.85	0.457	68.57	77.36
E2	8.40	0.339	0.654	0.545–0.752	66.67	67.31	0.332	58.54	74.47
AFC	6.50	0.820	0.965	0.902–0.993	88.89	93.15	0.814	86.49	94.44
SA	0.93	0.363	0.712	0.617–0.794	50.00	86.30	0.384	64.29	77.78
Total OV	3.27	0.655	0.879	0.803–0.934	86.11	79.45	0.612	67.39	92.06
Stroma_OV	1.81	0.640	0.872	0.795–0.928	75.00	89.04	0.645	77.14	87.84
Total_MG	33.70	0.406	0.668	0.571–0.755	69.44	71.23	0.380	54.35	82.54
Total_VI	3.49	0.292	0.669	0.573–0.756	33.33	95.89	0.342	80.00	74.47
Stroma_VI	3.77	0.315	0.687	0.591–0.773	34.29	97.26	0.373	85.71	75.53
Total_FI	24.03	0.350	0.689	0.593–0.774	58.33	76.71	0.345	55.26	78.87
Stroma_FI	24.08	0.324	0.687	0.591–0.773	57.14	75.34	0.317	52.63	78.57
Total_VFI	0.765	0.305	0.668	0.572–0.756	33.33	97.26	0.362	85.71	74.74
Stroma_VFI	0.875	0.315	0.685	0.588–0.771	34.29	97.26	0.373	85.71	75.53
PSV	13.01	0.349	0.685	0.589–0.771	52.78	82.19	0.359	59.38	77.92
EDV	5.98	0.351	0.698	0.603–0.782	66.67	68.49	0.326	51.06	80.65
Multi-parameter ultrasound		0.807	0.973	0.914–0.996	91.67	89.04	0.779	80.49	95.59
Ultrasound–serum combined index		0.942	0.995	0.949–1.000	100.00	94.23	0.929	92.11	100.00

Logistic regression (forward: LR method) screening.

Multi-parameter ultrasound: AFC + total_FI.

Ultrasound–serum combined index: AFC + stroma_VI + AMH + FSH.

**Table 4:** DeLong test for differences in area under the ROC curve between different indicators (*n* = 109).

Comparison	ΔAUC	SE	95% CI	*Z*-statistic	*p*-value
AFC vs. AMH	0.009	0.022	−0.034 to 0.052	0.421	0.673
AFC vs. multi-parameter ultrasound	0.007	0.005	−0.003 to 0.019	1.363	0.173
AFC vs. ultrasound–serum combined indicator	0.029	0.015	0.0004–0.059	1.991	**0.046**

Statistical significance was defined as *p* < 0.05.

AFC, antral follicle count; AMH, anti-Müllerian hormone.

The meaning of the bold value provided in Table (*p* = 0.046) was a statistically significant difference between AFC and the ultrasound-serum combined indicator.

### LASSO regression analysis

To address concerns regarding overfitting and multicollinearity among study variables, a LASSO regression analysis was employed to screen the 13 ultrasound variables with statistically significant inter-group differences from Table 2. A random number seed was set, the age variable was retained in the model, cross-validation was selected, and the *λ* value was chosen using a standard error rule. Simultaneously, the independent variables were standardized. The results of the LASSO regression analysis revealed five optimal variables: AFC, stromal OV, total VI, total FI, and EDV (Figure 3a). Notably, AFC exhibited the highest coefficient weight among them.

### Univariate and multivariate logistic regression analyses

Results of the univariate logistic analysis consistently demonstrated statistical significance across all variables: age (OR = 1.212, 95% CI:1.107–1.382, *p* < 0.001), stromal OV (OR = 0.360, 95% CI: 0.239–0.544, *p* < 0.001), EDV (OR = 0.735, 95% CI: 0.594–0.909, *p* = 0.005), total VI (OR = 0.919, 95% CI: 0.866–0.975, *p* = 0.005), total FI (OR = 0.843, 95% CI: 0.751–0.946, *p* = 0.004), and AFC (OR = 0.652, 95% CI: 0.544–0.783, *p* < 0.001). In the multivariate logistic analysis, AFC (OR = 0.596, 95% CI: 0.442–0.804, *p* < 0.001) and total FI (OR = 0.853, 95% CI: 0.737–0.988, *p* = 0.033) emerged as statistically significant factors (Table 5).

**Table 5:** Univariate and multivariate regression analyses.

Index	Univariable	Multivariable
Age	1.212 (1.107–1.328, *p* < 0.001)	1.140 (0.942–1.380, *p* = 0.179)
Stroma_OV	0.360 (0.239–0.544, *p* < 0.001)	1.101 (0.476–2.545, *p* = 0.823)
EDV	0.735 (0.594–0.909, *p* = 0.005)	0.769 (0.511–1.158, *p* = 0.209)
Total_VI	0.919 (0.866–0.975, *p* = 0.005)	0.965 (0.874–1.065, *p* = 0.479)
Total_FI	0.843 (0.751–0.946, *p* = 0.004)	0.853 (0.737–0.988, *p* =0.033)
AFC	0.652 (0.544–0.783, *p* < 0.001)	0.596 (0.442–0.804, *p* < 0.001)

OV, ovarian volume; EDV, end-diastolic velocity; VI, vascularization index; FI, flow index; AFC, antral follicle count; OR, odds ratio; CI, confidence interval.

### Ultrasound parameters between the left and right ovaries in the DOR group

The comparative analysis of DOR parameters between the left and right ovaries revealed significant statistical differences in total OV (*Z* = −2.467, *p* = 0.014) and stromal OV (*Z* = −2.465, *p* = 0.014), as well as in the PI (*t* = −2.348, *p* = 0.025) and RI (*t* = −2.605, *p* = 0.013) of the ovarian interstitial arteries. However, no statistically significant variances were found in the remaining ultrasonographic indicators (all *p* > 0.05) (Table 6).

**Table 6:** Comparison of ultrasound parameters between the left and right ovaries in the DOR group.

Index	Left ovary	Right ovary	*p*
Total OV (cm^3^)	3.53 (2.39, 4.28)	4.09 (2.76, 5.71)	0.014*
Stroma-to-antrum ratio (S/A)	0.92 ± 0.11	0.92 ± 0.08	0.990
AFC	3.00 (1.00, 3.75)	2.50 (1.00, 5.00)	0.489
Total MG (%)	34.03 ± 6.04	34.71 ± 5.74	0.529
Total VI (%)	6.62 (1.74, 13.36)	6.76 (2.83, 18.16)	0.060
Total FI (%)	21.79 ± 6.79	24.14 ± 6.41	0.125
Total VFI (%)	1.61 (0.34, 3.32)	1.60 (0.66, 5.10)	0.054
Stromal OV (cm^3^)	3.08 (2.25, 3.84)	3.50 (2.70, 4.91)	0.014*
Stromal MG (%)	35.15 ± 5.98	36.21 ± 5.37	0.310
Stromal VI (%)	7.08 (1.94, 13.92)	7.54 (3.25, 18.18)	0.067
Stromal FI (%)	21.94 ± 6.88	24.07 ± 6.45	0.172
Stromal VFI (%)	1.67 (0.36, 3.55)	1.78 (0.68, 5.10)	0.063
PSV (cm/s)	13.19 ± 5.22	14.72 ± 5.84	0.060
EDV (cm/s)	5.99 ± 2.83	5.83 ± 2.41	0.759
PI	0.89 ± 0.28	1.02 ± 0.38	0.025*
RI	0.54 ± 0.12	0.58 ± 0.11	0.013*

Values are presented as mean ± standard deviation. Statistical significance is indicated by *(*p* < 0.05) and ** (*p* < 0.01).

OV, ovarian volume; S/A, stroma-to-antrum ratio; AFC, antral follicle count; MG, mean gray; VI, vascularization index; FI, flow index; VFI, vascularization flow index; PSV, peak systolic velocity; EDV, end-diastolic velocity; PI, pulsatility index; RI, resistance index.

### Decision curve analysis comparing the net benefit of PRE_AFC and PRE_LASSO models across a range of threshold probabilities

**Figure 4:**
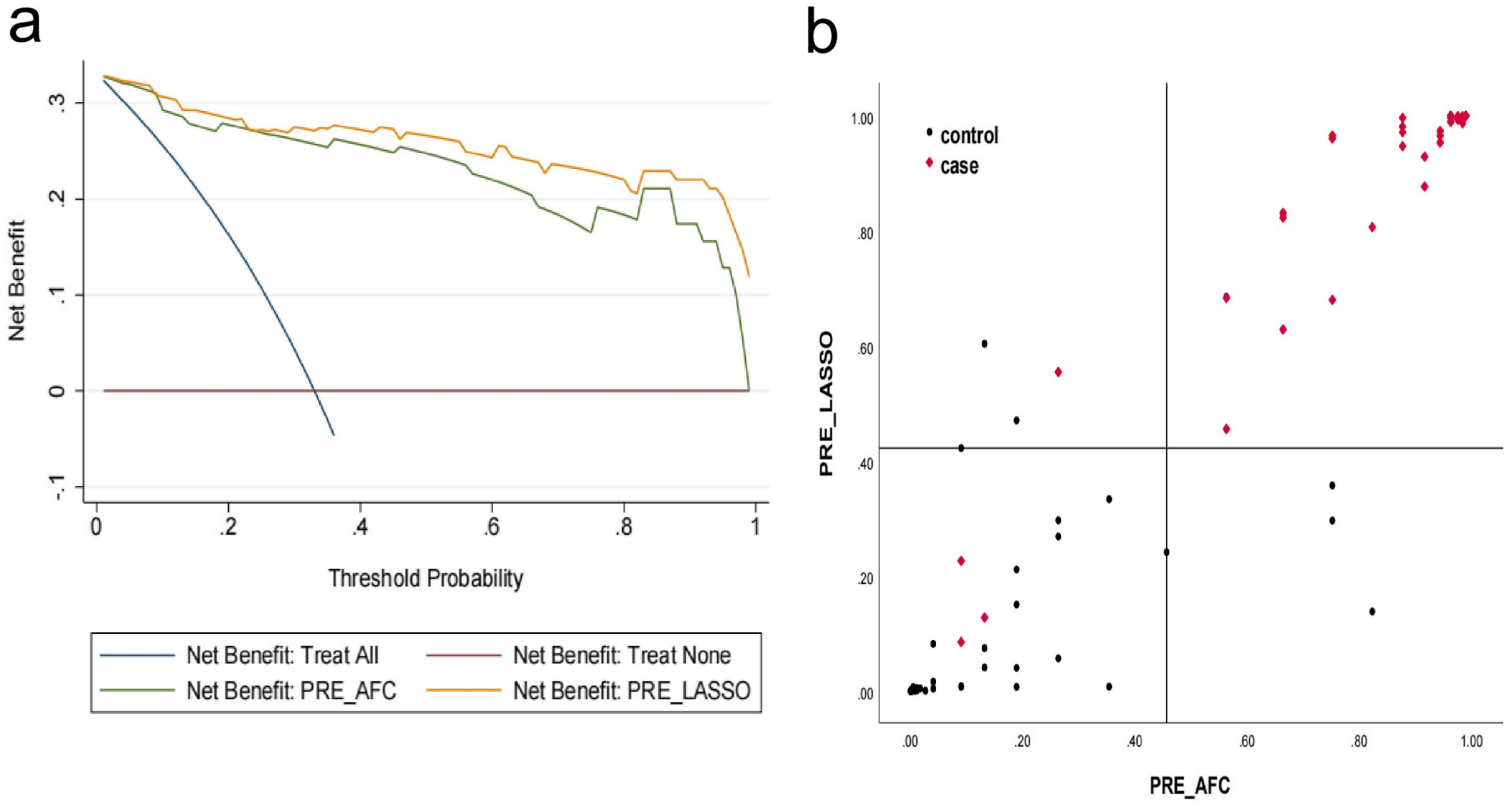
Decision curve analysis (DCA) and scatter plot of net reclassification improvement (NRI) of PRE AFC and PRE LASSO models. **(a)** The *x*-axis represents the threshold probability for clinical intervention, and the *y*-axis represents the net benefit. The blue line indicates the net benefit of treating all patients; the purple line indicates the net benefit of treating no patients (serving as the baseline of zero net benefit). The green curve represents the net benefit of the PRE_AFC model, and the orange curve represents the net benefit of the PRE_LASSO model. The orange curve (PRE_LASSO) lies above the green curve (PRE_AFC), indicating superior clinical utility. **(b)** The *x*-axis shows predicted probabilities from the PRE_AFC model, and the *y*-axis shows predicted probabilities from the PRE_LASSO model. Red diamonds denote patients with events (case group), and black dots denote patients without events (control group). Red diamonds (cases) are mostly upward-reclassified, while black dots (controls) are mostly downward-reclassified, confirming better risk stratification by PRE_LASSO.

The decision curve analysis (DCA) demonstrates that the PRE_LASSO model consistently outperforms the PRE_AFC model in net benefit across most threshold ranges and demonstrates superior clinical application value (Figure 4a).

### Scatter plot of net reclassification improvement illustrating the reclassification of individual patient risk by the PRE_LASSO model relative to the PRE_AFC model

The scatter plot of net reclassification improvement (NRI) demonstrated that most case patients had their risk elevated while control patients had their risk reduced, with NRI + =0.028, and the PRE_LASSO model demonstrated superior risk stratification capability (Figure 4b).

## Discussion

With the increasing incidence of infertility, assisted reproductive technology (ART) has become an effective treatment, so accurate assessment of ovarian reserve function is a key factor influencing its success rate. When ovarian function declines, the secretion of inhibin decreases and FSH levels increase significantly, and consequently, there is a rise in the FSH/LH ratio. In our study, the FSH and FSH/LH ratios in the DOR group were significantly higher than those in the normal group, with statistically significant differences. However, due to the fluctuation of basal hormone levels throughout the menstrual cycle, the timing of testing is strictly limited, and the elevation of FSH occurs relatively late, resulting in suboptimal accuracy and sensitivity when basal hormone levels alone are used to predict ovarian reserve function. AMH is primarily secreted by granulosa cells of preantral and antral follicles, reflecting the number of early follicles and playing an important regulatory role in follicular growth and development. Some foreign scholars believe that AMH is the most reliable indicator for predicting ovarian reserve and reproductive potential and can independently reflect ovarian reserve (11). In this study, the AUC of AMH in predicting ovarian reserve function was higher than that of FSH, with higher sensitivity and specificity. The relative stability of AMH during the menstrual cycle (12) and its detection not being affected by menstrual timing make AMH more suitable for clinical application and promotion.

While previous studies have highlighted basal hormone levels and serum AMH as reliable indicators of ovarian reserve (13, 14), currently, basal hormones and serum AMH alone have limitations in single ovary evaluations, restricting their clinical utility (14). Ovarian imaging, particularly with advancements in ultrasound technology such as transvaginal 3D ultrasound, has gained prominence due to its simplicity, non-invasiveness, intuitiveness, and safety (15). This method allows for the objective evaluation of ovarian morphology and quantitative measurement of various indicators, proving valuable in assessing ovarian reserve function, particularly in assisted reproductive technology (ART) (16). As ovarian reserve declines, the number of antral follicles decreases, ovarian volume diminishes, and blood flow to the ovarian stroma reduces. Previous studies using 3D ultrasound have employed quantitative indicators, such as OV, AFC, VI, FI, VFI, and PSV, to monitor changes in ovarian morphology, function, and blood flow dynamics (17–19). In this study, OV, AFC, VI, FI, VFI, PSV, and EDV were significantly lower in the DOR group than in the normal group, indicating high sensitivity and specificity for early DOR prediction. Furthermore, LASSO regression analysis identified age, AFC, stromal OV, total VI, total FI, and EDV as the optimal variables. Among these variables, the AFC coefficient weight was the highest, indicating significant diagnostic value. Cross-validation on two dimensions of clinical decision value (DCA) and risk stratification capability (NRI) demonstrated that the PRE_LASSO model outperformed the PRE_AFC model, offering both higher clinical net benefit and more accurate patient risk stratification to guide clinical decision-making. Studies by Mutlu et al. (20) and Lee et al. (21) have similarly demonstrated the superior predictive value of AFC compared to other ultrasound parameters and hormonal markers. Our findings also confirm that 3D ultrasound parameters, particularly AFC, are robust predictors of ovarian reserve. These results highlight the promising potential of 3D ultrasound techniques to improve the precision of ovarian reserve assessments.

Some studies have found that, in early DOR patients, the right ovary’s OV is significantly larger, with increased levels of AFC, VI, VFI, and FI (22, 23). Our study compared various 3D ultrasound indicators between the left and right ovaries in the DOR group. The right ovary showed larger OV, PI, and RI values than the left ovary, likely due to anatomical differences in venous drainage (24). Furthermore, differences in arterial supply to the bilateral ovaries could also contribute to these variations. The bilateral ovarian arteries usually arise directly from the abdominal aorta, but unlike the right ovarian artery, the left ovarian artery can also originate from the renal artery and may have a longer course (25–27). These vascular differences could lead to variations in ovarian volume and perfusion indices, further explaining the observed disparities between the left and right ovaries. This study demonstrated that 3D ultrasound has greater value in evaluating the function of a unilateral ovary.

However, these studies predominantly focused on overall ovarian metrics without distinguishing between the ovarian stroma and the entire ovarian volume (28, 29). In contrast, our study utilized advanced technologies such as automated follicle measurement and superb microvascular imaging to separately assess both overall and stromal OV, VI, FI, and VFI. This approach not only captures the comprehensive ovarian morphology and hemodynamic changes but also provides a more detailed reflection of the stromal morphology, function, and blood flow dynamics (30). By including these stromal-specific ultrasound indicators, our study offers a finer resolution of ovarian health assessment, potentially leading to more accurate and individualized diagnostics than previous research that did not consider these differentiated metrics (31, 32).

According to the 2015 consensus of the American Society for Reproductive Medicine, AMH and AFC are the most promising predictive factors for ovarian reserve function (33). Our study supports this finding, showing that AFC (AUC = 0.965, 95% CI: 0.902–0.993) is the most valuable ultrasound indicator for diagnosing DOR, followed by ovarian volume (OV). This study indicates that an AFC less than 6.50 is the optimal cutoff value for clinical application, consistent with findings suggesting that a bilateral ovarian AFC of less than 5–7 indicates DOR (9, 34). Previous studies have predominantly focused on the impact of total OV on ovarian reserve function. Gibreel et al. (35) proposed that an OV less than 3 cm^3^ indicates reduced ovarian reserve function. Our study suggests that total OV less than 3.27 cm^3^ and stromal OV less than 1.81 cm^3^ may indicate reduced ovarian reserve function, allowing for earlier prediction of DOR. A multivariate regression analysis further revealed that, except for AFC, total FI was also a risk factor for ovarian reserve function. However, this is precisely the advantage of three-dimensional transvaginal ultrasound.

The multi-parameter ultrasound indicator for diagnosing DOR resulted in an AUC of 0.973, with a sensitivity of 91.67% and a specificity of 89.04%. This diagnostic efficiency is higher than using AFC or OV independently but comparable to AMH. Combining hormonal and ultrasound examinations enhances sensitivity and specificity, improves diagnostic efficiency, reduces misdiagnosis, and provides a more accurate assessment of ovarian reserve function (23, 36).

In addition to the commonly used ultrasound indicators, we also measured new ones—S/A ratio and MG—to assess ovarian reserve function. The S/A ratio, the ratio of stromal OV to total OV, indicates a larger stromal volume and fewer antral follicles (37). MG, representing ovarian echogenicity, increases as the number of antral follicles decreases and the stromal proportion increases (38). Thus, MG can more intuitively display the intensity of echoes within the ovary, indirectly reflecting ovarian reserve function. Previous studies have used the stromal ovarian volume ratio and echogenicity to study PCOS. Research by Battaglia et al. (39) suggests that the stromal ovarian volume ratio can serve as a new diagnostic criterion for PCOS, while Christ et al. (40) found that follicle count is a better ultrasound marker for PCOS than ovarian stromal evaluation. In our study, the S/A ratio and MG were higher in the DOR group compared to the normal group, indicating a higher proportion of ovarian stroma, enhanced echogenicity, and reduced antral follicle count. Although the S/A ratio and MG were only mildly negatively correlated with serum AMH, their diagnostic efficiency for ovarian reserve function was lower than that of AFC and OV. Further research with larger sample sizes is needed to determine their ultimate diagnostic value.

While our study offers promising insights, it has several limitations. The retrospective design and single-center setting may limit the generalizability of the results. Although we recognize the limitations related to the relatively small sample size and the potential risk of model overfitting, we attempted to address this issue by employing the bootstrap method in LASSO regression for internal validation to assess model stability. However, the bootstrap sampling results were unsatisfactory due to the small sample size. Future research should focus on larger, multicenter, prospective studies to validate these findings. Automatic follicle measurement and superb microvascular imaging in transvaginal 3D ultrasound have certain limitations in identifying antral follicles in cases of far-field ovaries or when ovarian ultrasound images are unsatisfactorily displayed. Some follicles may be misidentified, and manual adjustment by the operator is still required. The differences in these subjective operations may lead to deviations in some measurement results. In this study, all scans were performed by a single doctor, which reduced variability but prevented the evaluation of inter-observer reproducibility. Therefore, research on inter-observer reproducibility can be conducted in future studies. However, discrepancies between our results and those of other studies may arise from differences in study populations, measurement techniques, and criteria for defining DOR. Additionally, variations in age distributions, ethnic backgrounds, and clinical settings can influence study outcomes (41). The precision of ultrasound measurements can vary based on the equipment used and the expertise of the operators (3, 42). In the future, the impact of other confounding factors on ovarian reserve should be explored.

## Conclusion

In conclusion, combining transvaginal 3D ultrasonography with superb microvascular imaging significantly improves the precision of ovarian reserve assessments. This approach provides valuable insights into ovarian morphology and vascularization, enhancing diagnostic accuracy for DOR.

## Data Availability

The original contributions presented in the study are included in the article/supplementary material, further inquiries can be directed to the corresponding authors.
